# Supplementary material for: A Critical View of the Application of the APEX Software (Aqueous Photochemistry of Environmentally-Occurring Xenobiotics) to Predict Photoreaction Kinetics in Surface Freshwaters
Source: Molecules. 2019 Dec 18;25(1):9. doi: 10.3390/molecules25010009 (PMC7017383; doi:10.3390/molecules25010009)
Supplement: Supplementary file 1 [file molecules-25-00009-s001.zip › 3.2.4_gcc-4.4.0.zip!/3.2.4_gcc-4.4.0/3.2.4_gcc-4.4.0/doc/octave/html/interpreter/Getting-Started.html]

Getting Started - Untitled


Next: Data Types,
Previous: Introduction,
Up: Top


---

## 2 Getting Started

This chapter explains some of Octave's basic features, including how to
start an Octave session, get help at the command prompt, edit the
command line, and write Octave programs that can be executed as commands
from your shell.

- Invoking Octave from the Command Line- Quitting Octave- Getting Help- Command Line Editing- Errors- Executable Octave Programs- Comments
